# Feeding levels during early gestation in a group-housing system for primiparous sows: impact on piglet birthweight and litter uniformity

**DOI:** 10.5713/ab.24.0415

**Published:** 2024-08-26

**Authors:** Natchanon Dumniem, Thomas D. Parsons, Padet Tummaruk

**Affiliations:** 1 Department of Obstetrics, Gynaecology and Reproduction, Faculty of Veterinary Science, Chulalongkorn University, Bangkok 10330, Thailand; 2 Swine Teaching and Research Unit – New Bolton Center, School of Veterinary Medicine, University of Pennsylvania, Kennett Square, PA 19348, USA; 3 Centre of Excellence in Swine Reproduction, Chulalongkorn University, Bangkok 10330, Thailand

**Keywords:** Backfat, Electronic Sow Feeder, Gestation, Group Housing, Loin Muscle

## Abstract

**Objective:**

The current study investigated the impacts of different feeding regimes during early gestation on conception rate, litter traits, piglet birthweight, and litter uniformity in primiparous sows.

**Methods:**

In total, 108 primiparous sows were inseminated and assigned to either a standard (1.9±0.5 kg/d, S) or high (2.9±0.8 kg/d, H) feeding levels during the first 35 days of gestation. The feeding regimes were categorized based on periods of gestation: 1 to 3, 4 to 15, and 16 to 35 days, resulting in four groups: standard-standard-standard (SSS, n = 26), standard-standard-high (SSH, n = 28), standard-high-high (SHH, n = 28), and high-high-high (HHH, n = 26). Afterwards, sows were placed into a group-housed system equipped with electronic sow feeders. The sows were weighed and assessed for backfat thickness and loin muscle depth at 0 and 35 days of gestation. At farrowing, data were collected on the total number of piglets born per litter, piglet birthweights, and the coefficient of variation of piglet birthweights.

**Results:**

On average, sows gained 22.5±21.6 kg during the first 35 days of gestation, showing a positive correlation with backfat gain (r = 0.954; p = 0.006). The backfat gain in the HHH group was higher than in the SSS (p = 0.016) and the SSH groups (p = 0.023), but did not differ from the SHH group (p = 0.684). Conception rates did not show differences among the feeding regimes (p>0.05). Individual piglet birthweights in the HHH group were higher than those in the SSH group (p<0.001). Likewise, the percentage of piglets with birthweights <1,000 g in the HHH group was lower than that in the SSH group (p<0.001). However, the variation of piglet birthweight did not differ among the groups (p>0.05).

**Conclusion:**

Increasing feeding levels in primiparous sows in a group-housed system during early pregnancy can effectively restore their body condition without any detrimental effects on subsequent litters.

## INTRODUCTION

In the early stages of gestation, sows allocate their energy towards various critical processes, including luteal formation, optimizing the uterine environment, embryo implantation, and supporting embryonic development [1]. These processes require significant energy expenditure for maintaining pregnancy. The uterine capacity for embryo attachment is a key determinant regulating placentation. The placenta acts as the primary conduit for nutritional transfer from mother to fetus via the uteroplacental circulation, thereby regulating fetal weight variation [2,3]. Maternal undernutrition negatively impacts placental efficiency, impairing blood supply and causing heterogeneity in fetal development, leading to piglet birthweight variation [2]. Increasing maternal feeding levels during early gestation can expand the functional space of the uterus and thus enhance embryo development [1]. However, restricted feeding during early pregnancy is a common practice in the swine industry based on studies from the 1990s that hypothesized high feed intake during early gestation might increase hepatic clearance of circulating progesterone, leading to early embryonic loss [4]. Conversely, recent research has shown that increasing feeding levels during early gestation can improve luteal tissue formation and enhance progesterone secretion from the corpus luteum [5,6]. It is well established that the serum progesterone from nutritional-induced is an important mediator for regulating the uterine environment and improving embryo survival [7]. For instance, gilts fed 2.8 kg of feed per day post-mating had higher serum progesterone concentrations and increased pulsatile secretion compared to those fed 1.5 kg per day [7]. Additionally, gilts receiving a post-mating diet at 1.5×the maintenance level exhibited a higher embryonic survival rate compared to those receiving 1.0×the maintenance level—88.4% and 77.8%, respectively —resulting in a greater number of conceptuses at day 25 of gestation, i.e., 14.0 and 11.7, respectively [8].

Primiparous sows often experience a negative energy balance during lactation [9]. Excessive body weight and backfat loss during this period can adversely affect the subsequent reproductive performance of primiparous sows [9,10]. Recent studies indicate that weaned sows with poor body condition or significant energy reserve loss show compromised follicle development, reduced numbers of corpora lutea, and smaller conceptus size [11,12]. Ye et al [12] showed that sows experiencing high backfat and loin muscle loss during lactation exhibit decreased plasma progesterone levels on day 8 after ovulation. Schenkel et al [10] demonstrated that primiparous sows losing more than 8% of their body weight or more than 9% of their protein mass during lactation had smaller litter sizes compared to sows losing less body weight or protein mass during lactation. In tropical climates, this problem is worsened by severe heat stress conditions and reduced sow appetites during the lactation period [13]. A previous study conducted in a commercial swine herd in tropical regions revealed that 43.9% of sows experience a loss of more than 10% of their backfat during the lactation period [14]. Primiparous sows typically exhibit greater backfat loss during lactation compared to multiparous sows [15]. Therefore, sufficient energy intake during early gestation is crucial for replenishing depleted body reserves from lactation, a process that cannot be fully compensated for within the wean-to-service interval [16]. As a result, current nutritional practices in the industry may not adequately support the successful implantation and development of early swine embryos [5,6]. This challenge is particularly pronounced in primiparous sows due to their smaller size and lower feeding capacity [15]. Thus, restricted feeding programs during early gestation may not sufficiently restore body reserves, support pregnancy maintenance, or reduce variation in subsequent piglet birthweights.

Simultaneously, increasing societal concerns regarding livestock welfare underscore the shift from traditional confined stalls to group housing systems [17]. In practice, electronic sow feeders are employed in these systems to provide precise feeding tailored to individual sows’ energy and nutrient needs [17]. However, in large dynamic group-housing systems, aggression, stress, and injuries resulting from the establishment of social hierarchies and competition for feed resources can disrupt sows’ ability to maintain pregnancy [18]. Consequently, competition for feed access and stress responses can curtail feeding time, reduce voluntary feed intake by sows [19], and thereby increase the risk of body weight loss during early gestation. Verdon et al [19] observed that dominant sows spent more time near the feeding area compared to submissive sows, leading to instances where some sows had no access to feed. Variability in feed intake among group members can intensify the impact of housing systems on reproductive performance during early gestation [20]. The heightened activity levels in group housing systems, combined with aggression from social interactions, may impact sow access to feeders, thereby complicating feeding regime management. We hypothesized that increasing feeding levels during early gestation could mitigate sow body weight loss during lactation, enhance luteal function, and improve embryo development. Therefore, the present study aimed to examine the effects of various feeding regimes during early gestation on sow conception rate, body weight, backfat thickness, and loin muscle gain during the initial 35 days of gestation. Additionally, we assessed litter size, piglet birthweight, and litter uniformity in subsequent litters.

## MATERIALS AND METHODS

### Animals and experimental design

The study was conducted at a commercial swine breeding farm in the central region of Thailand, with a herd size of 5,000 sows, from February to June 2022. All animal procedures were approved by the Institutional Animal Care and Use Committee in accordance with Chulalongkorn University regulations and policies governing experimental animal care (approval no. 2131053). In total, 108 crossbred Canadian Landrace×Yorkshire primiparous sows were selected for the study based on displaying estrus within 7 days after weaning and being in good condition. During the first 35 days of gestation, sows were allocated to different feeding levels: standard (S; 1.0×maintenance energy level) or high (H; 1.5×maintenance energy level). The standard feeding level provided a daily diet of 1.9±0.5 kg/sow/d, as per the national research council guidelines [21]. Sows in the high feeding level group received a daily intake of 2.9±0.8 kg/sow/d. Specifically, during days 1 to 21 of gestation, sows in the standard group received 1.8 kg/sow/d, while those in the high-level group received 2.7 kg/sow/d. From day 22 to 35, feed allocation in both groups was adjusted based on sow backfat thickness at insemination. From day 36 to 108 of gestation, all sows received a consistent daily feed intake of 3.2 kg/sow/d. This was then reduced to 2.0 kg/sow/d from day 109 of gestation until farrowing. The dietary treatment duration was divided into three periods: 1 to 3 days, 4 to 15 days, and 16 to 35 days of gestation, corresponding to specific phases of embryo development including fertilization and early embryonic formation, maternal recognition and implantation, and embryonic growth and early placentation, respectively [3]. The gestating sows were categorized into four treatment groups based on their feeding regimes during these periods: standard-standard-standard (SSS, n = 26), standard-standard-high (SSH, n = 28), standard-high-high (SHH, n = 28), and high-high-high (HHH, n = 26). The commercial gestational diet used was corn-soybean-based and contained 11.3 MJ/kg metabolizable energy, 12.7% crude protein, 5.7% crude fiber, and 0.7% lysine (905 BTG; Betagro Public Co., Ltd., Lopburi, Thailand). The detailed sow nutrient intake across different feeding levels is presented in Table 1.

### Housing and general management

After weaning, all sows were housed in individual stalls (1.80 ×0.65 m) from weaning until insemination. Estrus detection was performed twice a day in the presence of a mature boar. Sows exhibiting estrus were inseminated twice, at 12 and 24 h after standing estrus, via intrauterine insemination using pooled semen from at least two boars. Each dose contained 3,000×10^6^ motile spermatozoa in an 80 mL volume. The average lactation length, number of weaned piglets in the previous lactation, and wean-to-service interval were 21.7±1.3 days, 10.9±4.3 piglets, and 5.8±1.0 days, respectively. Thereafter, sows were allocated to a dynamic group-housed system with a maximum density of 220 sows per pen. Mixing of sows into the pen occurred 6 h after insemination to prevent semen reflux during relocation. These processes were carried out over a two-week period, after which the pen became static. At the start of the experiment, 80 gestating sows were already present in the pen, allowing the experimental sows to establish their social hierarchy within the existing pen population. A gestational pen measured 22×22 m with a fully concrete floor, providing 2.2 m^2^ per sow. Neither enrichment nor bedding were provided. The pen was equipped with six electronic sow feeders (Nedap Velos, Groenlo, the Netherlands) and 24 nipples for *ad libitum* access to drinking water. Each feeding station measured 2.0×0.6 m and was placed adjacent to the pen. The feed monitoring system software (Pig Farming – Nedap Velos 2021.1, Groenlo, the Netherlands) was used to identify sows consuming less than 50% of their daily feed allocation at 10 AM each day before the system reset. These sows were then assisted in accessing the electronic sow feeders to give them another opportunity to consume their full daily feed allotment [22,23]. Sows were kept in the group-housed system from 1 to 109 days of gestation, then moved to the free-farrowing house about 7 days before farrowing. The management of the free-farrowing system in Thailand was previously described in Dumniem et al [24]. The farrowing process was monitored from a distance, allowing sows to farrow naturally. All sows were housed indoors, equipped with an evaporative cooling system and temperature control devices (DOL-532; SKOV A/S, Roslev, Denmark). The average daily indoor temperature and humidity during the experimental period were 29.7°C±1.5°C (range 26.1°C to 32.2°C) and 69.1%±10.6% (range 41% to 85%), respectively. Furthermore, the average maximum daily temperature inside the barn was 31.2°C±1.1°C (range 26.6°C to 33.6°C).

### Measurement and data collection

Feeding data as well as the daily feed intake of each individual sow were obtained from the software integrated with the electronic sow feeders at 12 PM daily. The data was recorded from days 1 to 35 of gestation, including both the total feed offered (kg/sow/d) and the daily feed intake (kg/sow/d). The average daily feed intake was calculated for each period (days 1 to 3, 4 to 15, and 16 to 35) as well as for the entire early gestation period (days 1 to 35). The proportion of feed intake and the cumulative days of missed feeding per sow (MISSED) were calculated. Missed feeding was defined as the event when sows did not visit the feed station within a single feed day. The proportion of feed intake was calculated by dividing the daily feed intake by the daily feed allocation and multiplying by 100.

On days 0, 4, 16, and 35 of gestation, the sows were weighed using a digital scale integrated with the electronic sow feeders. Backfat thickness and loin muscle depth were measured at the P2 position using real-time B-mode ultrasonography (HS-2200; Honda Electronics Co., Ltd., Toyohashi, Japan). The measurements were taken by placing a 3.5 MHz linear ultrasound probe at the back of the sows, at the last rib, approximately 6 cm away from the midline on both sides. Body weight gain (kg), backfat gain (%), and loin muscle gain (%) during days 1 to 35 of gestation were then calculated.

Pregnancy detection was performed on all sows on day 35 of gestation using a real-time B-mode ultrasonography device, and the conception rate was calculated. The conception rate was determined by dividing the number of confirmed pregnant sows by the total number of inseminated sows and multiplying by 100. At farrowing, the farrowing rate was calculated by dividing the number of sows that completed farrowing by the total number of inseminated sows and multiplying by 100. Data on the date of mixing, date of farrowing, and gestation length were recorded for each sow. Reproductive performance metrics, including the total number of piglets born per litter (TB), number of piglets born alive per litter (BA), number of stillborn piglets per litter, and number of mummified fetuses per litter, were determined. Newborn piglets were individually weighed at birth using a digital scale (SDS IDS701-CSERIES; SDS Digital Scale Co. Ltd., Yangzhou, China), and the coefficient of variation (CV) of piglet birthweight within the litter was calculated. The proportion of stillborn piglets per litter, proportion of mummified fetuses per litter, and proportion of piglets that had birthweight <1,000 g were also calculated.

### Statistical analyses

All data were analyzed using SAS software version 9.4 (SAS Institute Inc., Cary, NC, USA). Descriptive statistics were computed for continuous and categorical data using the MEANS and FREQ procedures, respectively. The effects of feeding regime on feeding performance were evaluated using the general linear model procedure. The models included feeding regime (SSS, SSH, SHH, and HHH) as a fixed effect. Similarly, reproductive performance was assessed using the same model as described earlier, with litter size as a covariate. For the analysis of conception rate and farrowing rate, the factor considered included feeding regime, using the generalized linear mixed model procedure with feeding regime as a fixed effect. Individual piglet birthweight was analyzed using the general linear mixed model procedure, with feeding regime as a fixed effect and sow identities included as a random effect. Additionally, the effects of feeding regime on sow body weight, backfat thickness, loin muscle depth, as well as body weight gain, backfat gain, and loin muscle gain during the first 35 days of gestation were analyzed using the general linear model procedure. The statistical models included the feeding regime as a fixed effect. Differences in means for each variable class were compared using the Tukey-Kramer test. Pearson’s correlation was used to examine associations among sow body weight, backfat thickness, loin muscle depth, body weight gain, backfat gain, and loin muscle gain during the first 35 days of gestation. A p-value of less than 0.05 indicated statistical significance, while 0.05<p<0.10 was considered indicative of a tendency towards significance.

## RESULTS

### Feed intake

**Figure 1:**
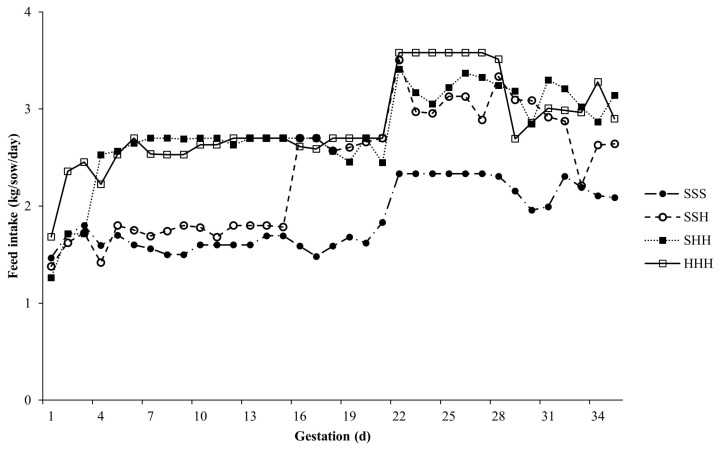
Feed intake during the early gestation period of primiparous sows according to feeding regimes: standard (S) = 1.8 to 2.8 kg/sow/d and high (H) = 2.7 to 4.2 kg/sow/d, from day 1 to day 35 of gestation in standard-standard-standard (SSS), standard-standard-high (SSH), standard-high-high (SHH), and high-high-high (HHH) groups.

**Figure 2:**
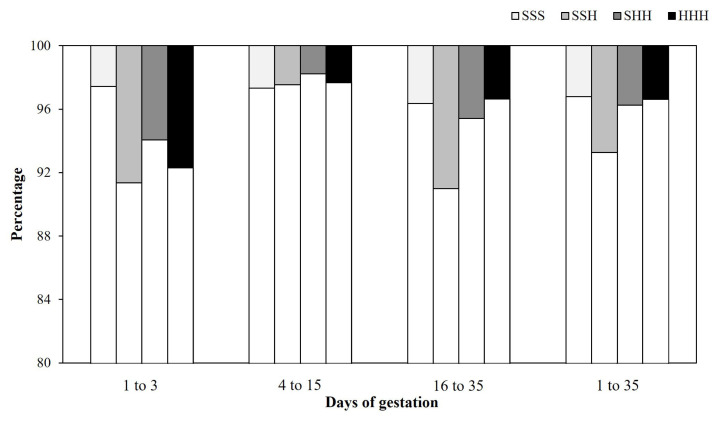
Distribution of sows that missed feeding (gradient bars) compared to sows that consumed all feed provision (white bars) during days 1 to 3, 4 to 15, 16 to 35 of gestation, and overall early gestation of primiparous sows according to feeding regimes: standard (S) = 1.8 to 2.8 kg/sow/d and high (H) = 2.7 to 4.2 kg/sow/d, from day 1 to day 35 of gestation in standard-standard-standard (SSS), standard-standard-high (SSH), standard-high-high (SHH), and high-high-high (HHH) groups.

Across groups, daily feed intake of sows during the first 35 days of gestation averaged 2.5±0.4 kg/sow/d and varied among individual sows from 0.0 to 3.1 kg/sow/d. The average daily feed intake during 1 to 3, 4 to 15, and 16 to 35 days of gestation were 1.8±0.5, 2.2±0.5, and 2.8±0.5 kg/d, respectively. The average daily feed intake of sows during the first 35 days of gestation classified by the four feeding regimes (SSS, SSH, SHH, and HHH) groups are presented in Figure 1. Across groups, the cumulative days of MISSED averaged 1.1±1.9 days. Figure 2 presents the distribution of sows that missed feeding compared to consuming all feed provision.

During the initial 3 days of gestation, sows in the HHH group exhibited higher feed intake compared to those in the SSS, SSH, and SHH groups (p<0.001; Table 2). Similarly, from days 4 to 15 of gestation, sows in the HHH group showed greater feed intake than those in the SSS (p<0.001) and SSH (p<0.001) groups, though their intake did not significantly differ from that of sows in the SHH group (Table 2). Between days 16 and 35 of gestation, sows in the HHH group consumed more feed compared to those in the SSS (p<0.001) and SSH (p = 0.018) groups, with no significant difference observed compared to the SHH group (Table 2). Additionally, sows in the HHH group had fewer cumulative days of MISSED during the first 35 days of gestation compared to sows in the SSH group (p = 0.050), but did not differ significantly from sows in the SSS and SHH groups (Figure 2).

### Sow body condition

Across groups, sows gained 22.5±21.6 kg in body weight, 20.8% in backfat thickness, and 19.8% in loin muscle depth during the first 35 days of gestation. Positive correlations were found between sow body weight gain and both backfat gain (r = 0.954; p = 0.006) and loin muscle gain (r = 0.569; p = 0.060). However, backfat gain was not associated with loin muscle gain during the first 35 days of gestation (r = 0.167; p = 0.145).

No differences were observed in body weight, backfat thickness, or loin muscle depth among sows from different feeding regime groups at the onset of the experiment (p>0.05). However, by day 35 of gestation, sows in the SHH group had higher backfat thickness compared to those in the SSS group (p = 0.016; Table 3). Additionally, sows in both the HHH and SHH groups had greater loin muscle depth on day 35 of gestation compared to those in the SSS and SSH groups (p< 0.05; Table 3). Backfat gain was higher in the HHH and SHH groups compared to the SSS and SSH groups (p<0.05; Table 3). Moreover, loin muscle gain during the first 35 days of gestation was greater in the HHH and SHH groups compared to the SSH group (p<0.05; Table 3).

### Conception rate and subsequent reproductive performance

Across groups, the conception rate and farrowing rate were 88.9% and 83.3%, respectively. The average TB was 14.2±2.1, BA was 13.2±2.3, and the proportions of stillborn piglets and mummified fetuses per litter were 5.3% and 2.0%, respectively. The average piglet birthweight was 1,417±351 g, with 9.5% of piglets weighing less than 1, 000 g, and the CV of piglet birthweight was 20.3%. There were no significant differences in conception rate or farrowing rate among the groups (Table 4). Similarly, TB, BA, the proportion of mummified fetuses per litter, and the CV of piglet birthweight did not differ among groups (Table 4). However, the proportion of stillborn piglets per litter was higher in the SSS and HHH groups compared to the SSH group (p<0.05; Table 4). The piglet birthweights in the HHH and SHH groups were greater than those in the SSH group (p<0.001) but did not differ significantly from the SSS group (Table 4). Additionally, the proportion of piglets with birthweights under 1,000 g was lower in the HHH and SHH groups compared to the SSH group (p<0.01; Table 4).

## DISCUSSION

In the present study, improving feeding levels involved not only increasing the feed quantity but also enhancing nutrient intake. According to Table 1, sows on the high feeding regime received 52% more nutrients compared to those on the standard feeding regime. This indicates that the high feeding levels reflect greater nutritional intake, not just increased feed quantity. The current study hypothesized that increasing feeding levels during early gestation could mitigate sow body weight loss during lactation, enhance luteal function, and improve embryo development. We found that increasing feed intake during the first 35 days of gestation from 1.9 kg/sow/d (SSS) to 2.9 kg/sow/d (HHH) did not increase sow body weight gain or loin muscle gain but did significantly improve backfat thickness gain. This suggests that adjusting the feeding regime during early gestation can alter some parameters of sow body condition. However, fertility and litter traits remained unchanged. Notably, the proportion of piglets with birthweight <1,000 g was significantly reduced in sows with an average daily feed intake of 2.8 kg/d (SHH) to 2.9 kg/d (HHH) compared to sows with an intake of 2.4 kg/d (SSH). Despite this, the within-litter variation in piglet birthweight was not significantly improved and ranged from 18.7% to 20.8%. This indicates that factors influencing piglet birthweight variation are multifaceted and that adjusting feed intake during early gestation alone is insufficient to address the issue fully.

The current findings did not support our hypothesis that increasing feeding levels during early gestation could enhance conception rates, farrowing rates, and reduce birthweight variation. The farrowing rate of primiparous sows observed in this study was relatively low (83.3%), consistent with previous findings under tropical conditions [25]. This indicates that poor fertility in sows under heat stress conditions remains a significant issue that needs further exploration [13]. In this study, the conception rate ranged from 78.6% in sows with limited feed allowance from days 0 to 15 of gestation to 96.4% in sows with limited feed allowance from days 0 to 3 of gestation. However, no statistical significance was observed due to the limited number of observations per group. Nevertheless, the observed trend of improved conception rates in primiparous sows with higher feed intake during the first 35 days of gestation suggests the potential for increasing feed intake during early gestation without significant detrimental effects.

Interestingly, sows that received a limited feed allowance of 2.4 kg/sow/d from days 0 to 15 of gestation had the highest cumulative days of missed feeding (2.0 days) and the lowest conception rate (78.6%). This suggests that in a group housing system, these sows may have failed to establish pregnancy due to refusing their daily feed allotment. Vargovic et al [26] found that multiparous sows in group housing, fed using an electronic sow feeding system, had an average of 3.6 missed feeding days and 5.4 days of feed intake below 70% throughout gestation, with most sows visiting the feed station only once a day. In our study, the cumulative days of missed feeding averaged 1.1 days across all groups and 2.0 days in the sows with limited feed allowance from days 0 to 15 of gestation, who had the lowest conception rate. Although severe feed restriction during early gestation is uncommon in general practices, inadequate nutrient intake post-insemination can suppress corpus luteum development [1,6]. Feed deprivation can delay luteal formation before implantation through the insulin and insulin growth factor-I pathways [1]. Consequently, maternal feed intake directly affects luteinizing hormone (LH) concentration, which regulates luteal function and embryo implantation [1].

In this study, primiparous sows supplemented with 1.5× maintenance level of feed during the first 35 days of gestation demonstrated potential for improving their body condition parameters without significant adverse effects on subsequent reproductive performances. In gilts, feeding at 1.2×maintenance level for the first 35 days of gestation led to a higher conception rate compared to those fed at 0.6×maintenance level, with rates of 88.9% vs 77.8% [27]. Additionally, embryos from the restricted feeding group expressed fewer genes related to developmental regulation on days 25 and 35 of gestation [27]. Similarly, Virolainen et al [28] found that increasing the energy level from 27 to 54 MJ/d for 35 days post-insemination improved LH concentration, leading to higher conception rates. Likewise, feeding gilts at 1.5×maintenance level after insemination increased the number of embryos and embryo survival rate on day 25 of gestation compared to feeding at 1.0×maintenance level [8]. Furthermore, Haen et al [29] observed higher LH pulses from days 11 to 21 after insemination in pregnant gilts compared to those that aborted. These findings highlight the benefits of increased feeding levels in supporting reproductive success by regulating reproductive hormones associated with pregnancy establishment during early gestation.

Other potential reasons for the poor conception rate in primiparous sows in a group housing system in this study could be attributed to increased acute stress from aggressive encounters, possibly due to the nature of the group housing system or farm management practices involving the assisting of sows to access to electronic sow feeders around the implantation period [20,22]. Stress during early gestation can have adverse effects on embryo survival [20]. Elevated cortisol levels can delay plasma estrone sulfate concentration, impacting maternal recognition [30]. Stress induced by feed deprivation can lead to delayed embryo development, reduced progesterone concentrations, and smaller placental size in sows [31]. Based on our hypothesis that current feeding practices during early gestation may be insufficient in a group housing system, where sows require more energy to cope with social hierarchy formation and increased daily activity, increasing feeding levels during this period could prove beneficial without disrupting pregnancy establishment.

In general, it is recommended to restrict feeding for sows during early gestation to 1.5 to 2.5 kg per sow per day [4,5]. However, when the feed allowance was increased to 2.9 kg/sow/d (HHH), sows were able to consume nearly all the provided feed, with an intake proportion exceeding 95%. This indicates that modern hyperprolific sows have a greater capacity for feed consumption than before. Additionally, these sows often experience significant body reserve depletion during lactation due to nursing large litters [32]. The body condition of sows at weaning significantly impacts their subsequent reproductive performance, particularly in primiparous sows with limited feeding capacity [15,32]. Weaned sows in a negative energy balance exhibited smaller follicle sizes and poorer zygote development [11], resulting in lower conception rates and fetal growth in subsequent gestations [33]. However, feeding management before insemination is constrained by the shortened wean-to-estrus interval, highlighting the need to compensate for body reserve loss by increasing feed during early pregnancy instead [5,34]. This study demonstrated that increasing feeding levels from 1.9 kg/sow/d to 2.8 to 2.9 kg/sow/d improved sow backfat thickness and loin muscle depth by day 35 of gestation. Specifically, sows fed 2.8 to 2.9 kg/d showed a 25.4% to 27.6% increase in backfat thickness and a 20.0% to 23.7% increase in loin muscle depth by day 35 of gestation compared to initial levels at insemination. These findings are consistent with previous studies demonstrating the benefits of increased feeding levels during early gestation [33,35]. This indicates that increasing feed intake during early gestation positively affects the replenishment of body reserves in primiparous sows after significant depletion during the previous lactation. However, in the present study, increased feeding did not result in a significant increase in sow body weight or body weight gain. In contrast, a previous study in Australia found that multiparous sows (parity numbers 2 to 8) with high lactational weight loss, which received a high feed allowance of 3.5 kg/d during the first 30 days of gestation, showed significantly higher weight gain (+6 kg) compared to sows that received a standard gestation feeding regime of 2.6 kg/d during the same period [36]. The differences between the present study and the previous one include the higher feed allowance during early gestation in the previous study (3.5 kg/d) compared to the present study (1.9 to 2.9 kg/d), and the higher parity numbers of sows used in the previous study. Primiparous sows must allocate more of their feed intake for body growth because they have not yet reached mature body weight, unlike multiparous sows. Therefore, the lower feed allowance and the use of only primiparous sows in the present study may have prevented a significant improvement in their body weight gain with feed allowances between 1.9 and 2.9 kg.

In the present study, litter size did not differ among the feeding regimes during early gestation, which is consistent with previous research [35]. However, Hoving et al [33] observed an increase of two piglets in primiparous sows fed 3.25 kg/d compared to those fed 2.5 kg/d from days 3 to 32 after insemination. These findings suggest that high feeding levels during early gestation do not adversely affect embryo survivability and litter size at subsequent farrowing. Furthermore, piglet birthweights were higher in sows that received 2.8 to 2.9 kg/d of feed during early gestation compared to those that received 2.4 kg/d. This contrasts with a previous study, which found no differences in the weight and viability of embryos in gilts fed either low (2.0 kg/d) or high (4.0 kg/d) diet levels for 7 days after insemination [37]. Therefore, the effects of feeding levels during early gestation on litter size remain controversial. In conclusion, the present study revealed that increasing feeding levels for primiparous sows in a group-housed system during early pregnancy effectively maintains their body condition without any detrimental effects on subsequent litters.

The limitation of the present study was the lack of data on sow body weight loss and their feed intake during the previous lactation. However, all body condition parameters, including body weight, backfat thickness, and loin muscle depth, were evaluated at the onset of the experiment, and no differences in these parameters were detected. This approach aimed to balance body conditions to solely evaluate the impact of different feeding regimes on sow litter performance. Under field conditions, some sows might have lost varying degrees of body reserves during the previous lactation, potentially leading to different reproductive outcomes. Therefore, for sows that lost excessive body reserves during the previous lactation, additional feeding regimes could be applied [1,33]. Under tropical conditions, one of the most common problems for lactating sows is insufficient feed intake to meet the nutrient requirements of lactation [13,15]. In practice, lactating sows are generally fed up to *ad libitum*, with an average daily feed intake of 5.8 kg/sow/d [15]. Reduced feed intake also increases sow body weight loss during lactation, decreases milk production, and lowers piglet weaning weight [38]. Therefore, lactation feed intake is clearly an important issue, especially with larger litters [13]. However, feeding regimes during the early gestation period in sows with different body weight loss during their previous lactation need to be carefully investigated further.

## Figures and Tables

**Table 1:** Sow nutrient intake in different feeding levels

Items	Standard feeding level	High feeding level
Feed allowance (kg/d)	1.9±0.5	2.9±0.8
Metabolizable energy (MJ/d)	21.5	32.8
Crude protein (g/d)	241.3	368.3
Crude fiber (g/d)	108.3	165.3
Lysine (g/d)	13.3	20.3

**Table 2:** Sow feeding performance during early gestation in feeding regimes (least-square means±standard error of the mean)

Variables	Feeding regimes^1)^				p-value

	SSS	SSH	SHH	HHH	
Average daily feed intake (kg/sow/d)					
Days 1 to 3	1.7±0.1^a^	1.6±0.1^a^	1.6±0.1^a^	2.3±0.1^b^	<0.001
Days 4 to 15	1.7±0.0^a^	1.8±0.0^a^	2.6±0.0^b^	2.6±0.0^b^	<0.001
Days 16 to 35	2.1±0.1^a^	2.8±0.1^b^	3.0±0.1^b^	3.1±0.1^b^	<0.001
Total	1.9±0.1^a^	2.4±0.1^b^	2.8±0.0^c^	2.9±0.1^c^	<0.001
Proportion of daily feed intake (%)					
Days 1 to 3	94.4	87.0	88.4	85.6	0.366
Days 4 to 15	97.0	95.6	97.3	96.9	0.870
Days 16 to 35	95.5^a^	90.1^b^	94.8^a^	95.8^a^	0.110
Total	96.4^a^	91.6^b^	95.0^ab^	95.1^ab^	0.089
Cumulative days of missed feeding	0.7±0.4^a^	2.0±0.4^b^	1.0±0.4^a^	0.9±0.4^a^	0.103

1) SSS, standard-standard-standard; SSH, standard-standard-high; SHH, standard-high-high; HHH, high-high-high.

a–c Different letters indicate significant difference (p<0.05).

**Table 3:** Body weight, backfat thickness, and loin muscle depth of sows during early gestation in feeding regimes (least-square means±standard error of the mean)

Variables	Feeding regimes^1)^				p-value

	SSS	SSH	SHH	HHH	
Body weight (kg)					
Day 0	192±5	186±5	190±5	193±5	0.802
Day 4	193±6	191±6	188±6	191±6	0.937
Day 16	180±10	193±9	196±9	199±10	0.512
Day 35	211±5	212±5	210±4	215±5	0.857
Backfat thickness (mm)					
Day 0	13.5±0.3	14.2±0.3	14.0±0.3	13.6±0.3	0.730
Day 4	13.6±0.6	14.5±0.3	14.0±0.3	14.0±0.3	0.845
Day 16	14.1±0.6	14.9±0.4	15.5±0.4	14.8±0.4	0.190
Day 35	15.4±0.7^a^	16.2±0.6^ab^	17.5±0.5^b^	16.7±0.6^ab^	0.049
Loin muscle depth (mm)					
Day 0	38.2±0.8	38.5±0.8	38.4±0.8	38.4±0.8	0.996
Day 4	39.5±0.7	40.2±0.7	38.8±0.7	39.4±0.7	0.562
Day 16	42.8±0.9	44.3±0.8	44.0±0.8	43.8±0.8	0.637
Day 35	44.9±0.6^a^	44.2±0.6^a^	47.0±0.6^b^	46.6±0.6^b^	0.003
Body weight gain (kg)	21.1±4.7	28.6±4.6	20.3±4.2	20.4±4.6	0.461
Backfat gain (%)	15.4^a^	16.6^a^	26.3^b^	23.9^b^	0.018
Loin muscle gain (%)	19.5^ab^	14.1^b^	23.2^a^	21.5^a^	0.040
Proportion of sows losing backfat (%)	22.7	18.2	11.5	9.1	0.587

1) SSS, standard-standard-standard; SSH, standard-standard-high; SHH, standard-high-high; HHH, high-high-high.

a,b Different letters indicate significant difference (p<0.05).

**Table 4:** Conception rate, farrowing rate, and reproductive performances in the second litter of sows in different feeding regimes from days 1 to 35 of gestation (least-square means±standard error of the mean)

Variables	Feeding regimes^1)^				p-value

	SSS	SSH	SHH	HHH	
Number of sows	23	22	27	22	
Gestation length (d)	115.9±0.4	115.3±0.4	115.3±0.4	115.5±0.4	0.832
Conception rate (%)	88.5	78.6	96.4	84.6	0.176
Farrowing rate (%)	84.6	75.0	92.9	80.8	0.159
Total number of piglets born per litter	13.5±0.8	15.0±0.8	13.4±0.7	14.6±0.7	0.616
Number of born alive piglets per litter	12.6±0.7	14.6±0.7	12.6±0.7	13.3±0.7	0.641
Proportion of stillborn piglets per litter (%)	6.1^a^	1.9^b^	4.0^ab^	6.5^a^	0.125
Proportion of mummified fetuses per litter (%)	1.4	0.8	1.6	1.9	0.899
Individual birthweight (g)	1,410±26^a^	1,322±26^b^	1,466±24^a^	1,449±23^a^	<0.001
Piglets with birthweight <1,000 g (%)	10.3^ab^	17.9^a^	6.5^b^	5.2^b^	<0.001
Coefficient of variance of piglet birthweight (%)	18.7	20.8	20.1	20.1	0.916

1) SSS, standard-standard-standard; SSH, standard-standard-high; SHH, standard-high-high; HHH, high-high-high.

a,b Different letters indicate significant difference (p<0.05).
